# PTK7 Faces the Wnt in Development and Disease

**DOI:** 10.3389/fcell.2017.00031

**Published:** 2017-04-05

**Authors:** Hanna Berger, Andreas Wodarz, Annette Borchers

**Affiliations:** ^1^Department of Biology, Molecular Embryology, Philipps-Universität MarburgMarburg, Germany; ^2^Department of Anatomy I, Molecular Cell Biology, University of CologneCologne, Germany; ^3^Cluster of Excellence - Cellular Stress Responses in Aging-Associated Diseases, University of CologneCologne, Germany; ^4^DFG Research Training Group, Membrane Plasticity in Tissue Development and Remodeling, GRK 2213, Philipps-Universität MarburgMarburg, Germany

**Keywords:** PTK7, Wnt signaling, planar cell polarity, cancer, neural tube defect, scoliosis

## Abstract

PTK7 (protein tyrosine kinase 7) is an evolutionarily conserved transmembrane receptor regulating various processes in embryonic development and tissue homeostasis. On a cellular level PTK7 affects the establishment of cell polarity, the regulation of cell movement and migration as well as cell invasion. The PTK7 receptor has been shown to interact with ligands, co-receptors, and intracellular transducers of Wnt signaling pathways, pointing to a function in the fine-tuning of the Wnt signaling network. Here we will review recent findings implicating PTK7 at the crossroads of Wnt signaling pathways in development and disease.

## Introduction

PTK7 (protein tyrosine kinase 7) is an evolutionarily conserved transmembrane receptor with a broad range of functions in tissue development and homeostasis. Originally identified as a gene upregulated in colon carcinoma cells and accordingly named colon carcinoma kinase 4 (CCK-4) (Mossie et al., 1995) it was later shown to affect various aspects of cell-cell communication and movement. PTK7 controls tissue morphogenesis and patterning by affecting cell polarity, migration as well as tissue regeneration and wound healing (Lu et al., 2004; Shnitsar and Borchers, 2008; Caddy et al., 2010; Lee et al., 2011; Lander and Petersen, 2016). Additionally its function in adult tissue homeostasis is demonstrated by the fact that misregulation of PTK7 expression correlates with development of cancer and its progression to metastasis in various cellular contexts (reviewed in Dunn and Tolwinski, 2016). Furthermore, mutations in PTK7 have been implicated in scoliosis and human neural tube closure defects, demonstrating its clinical relevance (Hayes et al., 2014; Wang et al., 2015; Grimes et al., 2016). Since the first publication on PTK7/CCK-4 (Mossie et al., 1995) more than 20 years ago over 120 publications have followed. Although its signaling function is still far from being understood, recent findings provide compelling evidence that PTK7 is a regulator of Wnt signaling pathways. In this review we will summarize recent findings and take a look at PTK7's function in distinct Wnt signaling pathways.

Secreted glycoproteins of the Wnt family are key regulators of development and disease. Wnt ligands regulate a wide range of processes including primary embryonic axis specification, organogenesis and stem cell proliferation. Further, deregulated Wnt signaling has been implicated in various diseases like colon and breast cancer, melanoma, and neurodegenerative disorders (MacDonald et al., 2009; Clevers and Nusse, 2012; Anastas and Moon, 2013; Inestrosa and Varela-Nallar, 2014). Wnt ligands activate distinct downstream signaling pathways, and historically the first described, ß-catenin-dependent, signaling cascade is referred to as the “canonical” Wnt signaling pathway, while later discovered, ß-catenin-independent pathways were termed “non-canonical.” Canonical Wnt signaling (Logan and Nusse, 2004; MacDonald et al., 2009) is activated by binding of the Wnt ligand to a receptor complex consisting of the seven-pass transmembrane Frizzled (Fz) receptor and the low-density lipoprotein receptor-related protein 6 (LRP6) (MacDonald and He, 2012). Wnt binding to the Fz/LRP6 receptor complex leads to inactivation of glycogen synthase kinase 3ß (GSK3ß) regulating various intracellular substrates. One of these is the transcriptional co-activator ß-catenin, which is phosphorylated and thereby targeted for proteasomal degradation. Thus, in the presence of Wnt ligands, ß-catenin is stabilized, enters the nucleus and regulates in combination with transcription factors of the Lef (lymphoid enhancer-binding factor) and Tcf (T cell factor) family the transcription of target genes. In contrast to canonical Wnt signaling, non-canonical Wnt signaling pathways encompass a complex network of signal transducers that do not activate ß-catenin, but use alternative modes of downstream signaling (reviewed in Niehrs, 2012). Here, we will focus on the planar cell polarity (PCP) pathway, as PTK7 has been implicated in its regulation.

The PCP pathway (Goodrich and Strutt, 2011; Yang and Mlodzik, 2015) determines the orientation of cells in the plane of an epithelium and is one of the best-characterized non-canonical Wnt signaling pathways. PCP was first described in *Drosophila*, where genetic screens discovered its function in the polarization of adult cuticular structures. According to mutant phenotypes showing wing hair polarity defects the genes *Frizzled* (Fz) and *Disheveled* (Dsh) were identified. Other core PCP proteins include the four-pass transmembrane protein Van Gogh (Vang, Strabismus), the atypical cadherin Flamingo (Fmi, Celsr) and intracellular components like Prickle (Pk) and Diego (Dgo). These proteins confer intra- and intercellular signaling, thereby aligning PCP in neighboring cells. Complementary studies in vertebrates revealed that these core PCP proteins are also required for the polarization of vertebrate tissues, like the orientation of hair follicles in the epidermis or the sensory hair cells in the inner ear (Montcouquiol et al., 2006; Simons and Mlodzik, 2008; Wallingford, 2012). Furthermore, these proteins are also involved in the polarized localization of cilia, microtubule-based protrusions that are found on the surface of most vertebrate cells and required for fluid movement during development and homeostasis (Wallingford, 2010; Wallingford and Mitchell, 2011). In addition to the polarization of tissues, loss of function studies using the mouse, zebrafish and *Xenopus* model systems demonstrated that PCP signaling also affects morphogenetic cell movements shaping the embryonic body. One of these is convergent extension, a cell movement whereby cells intercalate in a way that a tissue converges in one direction and extends in the perpendicular direction (Wallingford et al., 2002; Wallingford, 2012). Convergent extension is required to drive gastrulation and neural tube closure. Consequently, misregulation of PCP signaling leads to severe gastrulation and neurulation defects in mouse, zebrafish and *Xenopus* embryos. Since the discovery of vertebrate PCP phenotypes, these have also contributed to the identification of novel vertebrate regulators of PCP without previous knowledge of a *Drosophila* phenotype. One of these genes, which was identified by its mouse neural tube closure and inner ear hair polarity defect, is PTK7.

## PTK7 affects Wnt signaling pathways

Vertebrate PTK7 is according to the current criteria a bona fide PCP regulator. Using a mouse gene trap-screen for transmembrane proteins with a function in neural development, PTK7 mutants were identified showing a combination of severe neural tube closure and inner ear polarity defects (Lu et al., 2004). Based on this mutant phenotype, which is typical for known regulators of PCP (Hamblet et al., 2002; Curtin et al., 2003; Montcouquiol et al., 2003), as well as its genetic interaction with Vangl2, PTK7 was added to the list of vertebrate PCP regulators. Further functional studies using mouse, zebrafish and *Xenopus* confirmed a function for PTK7 in processes that are regulated by PCP signaling, including convergent extension movements during gastrulation, neurulation and Wolffian duct elongation, as well as neural crest migration and wound healing (Table 1). Surprisingly, although PTK7 appears to be a core regulator of vertebrate PCP, classical PCP phenotypes have so far not been reported for the *Drosophila* orthologs of PTK7, *off-track* (*otk*), and *off-track2* (*otk2*). These two genes, which are the result of a tandem gene duplication, function redundantly in the tubular morphogenesis of the male ejaculatory duct, leading to male sterility in the *otk, otk2* double mutant (Linnemannstons et al., 2014). Intriguingly, mesoderm-specific knock-out of PTK7 in the mouse resulted in tubular morphogenesis defects in the Wolffian duct, again leading to male sterility (Xu et al., 2016). In both cases, tubular morphogenesis defects upon loss of Otk/Otk2 or PTK7 may be caused by the failure to properly execute convergent extension movements. Thus, although the *Drosophila* mutants do not display the classical PCP defects, PTK7/Otk may play an evolutionarily conserved role in the regulation of cell movements.

**Table 1:** **PCP phenotypes upon PTK7 loss of function in vertebrates**.

**PCP phenotype**	**Process**	**Mutant**	**References**
Craniorachischisis	Neural tube closure	Mouse, hypomorphic mutant (*Ptk7^*XST87*^*)	Lu et al., 2004
		Mouse, *chuzhoi* mutant (insertion of MT1-MMP splice site)	Paudyal et al., 2010
Convergent extension defect	Neural tube closure	Mouse, hypomorphic mutant (*Ptk7^*XST87*^*)	Lu et al., 2004; Williams et al., 2014
		*Xenopus*, Morpholino knockdown	Lu et al., 2004; Wehner et al., 2011
		Zebrafish, maternal-zygotic mutant (*ptk7^*hsc9*^*)	Hayes et al., 2013
	Gastrulation	Mouse, hypomorphic mutant (*Ptk7^*XST87*^*)	Yen et al., 2009
		Zebrafish, maternal-zygotic mutant (*ptk7^*hsc9*^*)	Hayes et al., 2013
	Wolffian duct morphogenesis	Mouse, hypomorphic mutant (*Ptk7^*XST87*^*)	Xu et al., 2016
Impaired stereociliary bundle orientation	Development of the organ of corti	Mouse, hypomorphic mutant (*Ptk7^*XST87*^*)	Lu et al., 2004; Lee et al., 2012; Andreeva et al., 2014
		Mouse, *chuzhoi* mutant (insertion of MT1-MMP splice site)	Paudyal et al., 2010
Impaired neural crest migration	Neural crest migration	*Xenopus* Morpholino knockdown	Shnitsar and Borchers, 2008; Podleschny et al., 2015
Defective wound repair	Epidermal wound repair	Mouse, hypomorphic mutant (*Ptk7^*XST87*^*)	Caddy et al., 2010
Defect in cilia development	Development of ependymal cell cilia	Zebrafish, zygotic mutant (*ptk7^*hsc9*^*)	Grimes et al., 2016

The molecular mechanism by which PTK7 affects PCP signaling is currently unclear. However, as PTK7 interacts with Wnt ligands and known Wnt receptors (Table 2) it likely affects PCP by functioning as a Wnt receptor. This is also supported by the structure of PTK7, which is highly reminiscent of receptor tyrosine kinases. PTK7 consists of seven extracellular immunoglobulin domains, a transmembrane domain, and an evolutionarily conserved tyrosine kinase homology domain. The kinase homology domain of PTK7 lacks catalytic activity (Miller and Steele, 2000; Kroiher et al., 2001), but serves as an interaction site for intracellular signaling molecules like ß-catenin, Dsh, and Src (Shnitsar and Borchers, 2008; Puppo et al., 2011; Andreeva et al., 2014). PTK7 interacts with distinct Wnt receptors including Fz7, LRP6, and Ror2 (Peradziryi et al., 2011; Bin-Nun et al., 2014; Linnemannstons et al., 2014; Martinez et al., 2015; Podleschny et al., 2015), indicating that PTK7 affects canonical and non-canonical Wnt signaling pathways. This is also reflected by its evolutionarily conserved interaction with different Wnt ligands that are supposed to signal via both canonical and non-canonical pathways (Peradziryi et al., 2011; Linnemannstons et al., 2014; Martinez et al., 2015). While its requirement for PCP signaling is firmly established, the function of PTK7 in canonical Wnt signaling remains controversial. PTK7 has been reported to activate canonical Wnt signaling in the context of Spemann's organizer formation (Puppo et al., 2011) and the specification of posterior neural tissue (Bin-Nun et al., 2014) in *Xenopus* embryos. However, PTK7 inhibits canonical Wnt signaling in *Xenopus* double axis and luciferase reporter assays (Peradziryi et al., 2011). This was confirmed by *ptk7* mutant zebrafish, which showed an upregulation of ß-catenin target gene expression, suggesting that PTK7 functions in attenuating canonical Wnt signaling (Hayes et al., 2013). Conflicting results were also obtained analyzing the interaction of PTK7 with Wnt ligands using immunoprecipitation of overexpressed/tagged constructs. While we found interaction of PTK7 with canonical Wnt3a and Wnt8 but not non-canonical Wnt5a (Peradziryi et al., 2011) in *Xenopus* lysates, Martinez et al. observed an interaction with non-canonical Wnt5a, but not canonical Wnt1 (Martinez et al., 2015) in HEK293T cells. Some of these contradictions may be explained by receptor context. Using secreted proteins we showed that the extracellular domain of PTK7 requires the extracellular Fz7 domain for interaction with recombinant Wnt3a (Peradziryi et al., 2011). Conversely, Wnt5a binding may require the Ror2 co-receptor. Although Martinez et al. confirmed interaction of PTK7 and Wnt5a in cells that were depleted of Ror2 using a specific siRNA, there may still be sufficient endogenous Ror2 present to mediate binding. Thus, studies analyzing direct interaction of PTK7 and Wnt ligands are currently lacking. Furthermore, experiments testing Wnt binding of combinatorial PTK7 co-receptor complexes are required. As PTK7 is a versatile receptor interacting not only with Wnt co-receptors but also with plexin and VEGF receptors (reviewed in Peradziryi et al., 2012), the latter interactions may also contribute to tissue-specific differences. Thus, future research will have to elucidate how receptor context affects PTK7 signaling and its functions in distinct Wnt signaling pathways.

**Table 2:** **PTK7 interaction partners with a known function in Wnt signaling**.

**Interaction partner**		**Interaction domain**	**Biological context**	**References**
Wnt ligand	Wnt3a, Wnt8	Extracellular domain	*Xenopus* double axis assay	Peradziryi et al., 2011
	Wnt4	Unknown	*Xenopus* double axis assay	Peradziryi et al., 2011
	Wnt5a	Extracellular domain (Ig4-7)	*Xenopus* morphogenesis	Martinez et al., 2015
	Wnt2	Unknown	*Drosophila* male fertility	Linnemannstons et al., 2014
Wnt receptor	Fz1	Unknown	*Drosophila* male fertility	Linnemannstons et al., 2014
	Fz2	Unknown	*Drosophila* male fertility	Linnemannstons et al., 2014
	Fz7	Extracellular domain	*Xenopus* luciferase reporter assay	Peradziryi et al., 2011
	Ror2	Extracellular domain Ig1-7	*Xenopus* morphogenesis and neural crest migration	Martinez et al., 2015; Podleschny et al., 2015
	LRP6	Transmembrane domain	*Xenopus* posterior neural development	Bin-Nun et al., 2014
Intracellular Wnt components	Dsh	Kinase homology domain (via Rack1/ PKCδ1)	*Xenopus* neural crest migration and neural tube closure	Shnitsar and Borchers, 2008; Wehner et al., 2011
	β-catenin	Kinase homology domain	*Xenopus* Spemann Organizer formation	Puppo et al., 2011

## PTK7 and disease

As PTK7 has a crucial function in the regulation of Wnt signaling pathways known to be essential for embryonic development and homeostasis, mutations in the human PTK7 gene are likely of clinical relevance. PTK7 was identified as a gene upregulated in colon carcinoma cells and appears to be misregulated in a variety of cancers (Dunn and Tolwinski, 2016). Furthermore, PTK7 mutations have recently been implicated in the etiology of neural tube defects and scoliosis (Hayes et al., 2014; Wang et al., 2015). Here, we will briefly describe these respective disorders and look at the human PTK7 gene variants identified in this context as well as their functional implications.

The connection between PTK7 and cancer has so far mostly been deduced on the basis of up- or downregulation of PTK7 in a variety of cancer types. PTK7 levels were reported to be increased in esophageal (Shin et al., 2013), gastric (Lin et al., 2012), colorectal (Lhoumeau et al., 2015), breast (Gartner et al., 2014), intrahepatic bile duct (Jin et al., 2014), prostate (Zhang et al., 2014), and lung carcinoma (Chen et al., 2014), as well as liposarcoma (Gobble et al., 2011). In other cancer types PTK7 was shown to be downregulated, including lung squamous cell carcinoma (Kim et al., 2014), ovarian carcinoma (Wang et al., 2014) and metastatic melanoma (Easty et al., 1997). While the mechanistic contribution of PTK7 to the respective tumor phenotypes is unclear at present, the upregulation of PTK7 in many tumor types makes it an attractive tumor marker and therapeutic target. Indeed, the first PTK7 specific reagents with potential clinical applications have now been published, including a PTK7-specific fluorescently labeled aptamer for *in vivo* detection of tumor tissue (Calzada et al., 2017). Very interestingly, PTK7 has recently been established as a marker for normal colon stem cells (Jung et al., 2015) and as a marker for tumor initiating cells in triple-negative breast cancer, ovarian cancer and non-small cell lung cancer (Damelin et al., 2017). The authors of the latter study also developed a PTK7-targeted antibody-drug conjugate and showed that its application reduces tumor initiating cells and induces sustained tumor regressions, paving the way for a PTK7-directed anti-tumor therapy (Damelin et al., 2017).

Neural tube defects are among the most common human birth defects affecting 1 per 1000 live births and are caused by environmental as well as genetic factors (Wilde et al., 2014). PCP genes are likely among the genetic factors contributing to the etiology of human neural tube closure defects as loss of function mutants of PTK7, Vangl, Celsr, Fz, Dvl, and Scribble result in the most severe neural tube closure defects called craniorachischisis (Gerrelli and Copp, 1997; Kibar et al., 2001; Hamblet et al., 2002; Curtin et al., 2003; Murdoch et al., 2003; Lu et al., 2004; Wang et al., 2006), whereby the neural tube fails to close from the midbrain-hindbrain boundary to the base of the spine. Indeed, rare mutations with a predicted damaging role were identified for a number of PCP genes including Vangl1/2, Celsr1, Fzd6, Dvl2, Prickle, and Scribble (Kibar et al., 2007; De Marco et al., 2014). Furthermore, the analysis of a cohort of 473 patients with various forms of neural tube defects identified six rare PTK7 sequence variants (Wang et al., 2015). Interestingly, five of these mutations are located in the extracellular domain of PTK7, which serves as interaction site for Wnt ligands as well as Fz7 and Ror2 receptors (Table 2; Peradziryi et al., 2011; Martinez et al., 2015; Podleschny et al., 2015). Whether these interactions are affected in the potentially pathogenic sequence variants is currently unclear and functional validation assays testing their efficiency to rescue for example *Xenopus* or zebrafish loss of function phenotypes are still missing. Nevertheless, the extracellular domain was shown to be important for promoting PCP and inhibiting canonical Wnt signaling. In fact, deletion of the extracellular domain abolished PTK7's ability to inhibit canonical Wnt signaling in *Xenopus* reporter assays (Peradziryi et al., 2011). Conversely, a membrane-tethered PTK7 extracellular fragment was sufficient to rescue excess Wnt/ß-catenin signaling and PCP morphogenesis defects in maternal-zygotic *ptk7* mutant zebrafish (Hayes et al., 2013). Thus, these data point to PTK7 as a risk factor for neural tube closure defects and stress the functional importance of its extracellular domain.

In addition to neural tube defects, PTK7 has also been implicated in the pathogenesis of scoliosis, a complex genetic disorder characterized by a three-dimensional spinal curvature. Congenital scoliosis (CS) is apparent at birth and involves abnormal vertebrae development, while idiopathic scoliosis is diagnosed during adolescence and does not show vertebral malformations. *Ptk7* mutant zebrafish were recently discovered as a model for congenital and idiopathic scoliosis. Maternal-zygotic *ptk7* (*MZptk7*) mutant zebrafish exhibit vertebral abnormalities at larval stages, phenotypically resembling congenital scoliosis. Further, zygotic *ptk7* (*Zptk7*) mutants show late onset spinal curvatures consistent with the idiopathic form of scoliosis (Hayes et al., 2014). Analysis of maternal-zygotic mutants showed that PTK7 positively regulates PCP-dependent morphogenesis, while it attenuates ß-catenin-dependent canonical Wnt signaling (Hayes et al., 2013). Thus, segmentation and somite patterning are disturbed, likely causing the observed vertebral abnormalities. In contrast, zygotic *ptk7* mutants did not show defects in segmentation and somite patterning, but developed late spinal curvatures resembling idiopathic scoliosis (Hayes et al., 2014; Grimes et al., 2016). They showed defects in ependymal cell cilia development leading to irregularities in the cerebrospinal fluid (CSF) flow. Moreover, the brain ventricles revealed a severe hydrocephalus, a condition associated with loss of cilia function. Consistently, the number of motile cilia was reduced and if cilia were present they lacked the correct polarization. Transgenic reintroduction of wild-type PTK7 in motile ciliated cell lineages rescued all phenotypes, proving a specific function of PTK7 in motile ciliated cells. The authors hypothesized that impaired cerebrospinal fluid flow due to abnormal cilia function is most likely the cause of scoliosis in *ptk7*-deficient zebrafish (Grimes et al., 2016). The connection of PTK7 to scoliosis was further evidenced by the isolation of a novel PTK7 mutation from a single patient suffering from idiopathic scoliosis. This mutation, hPTK7^P545A^, exhibits a proline to alanine substitution in the sixth extracellular immunoglobulin domain thereby affecting PCP and canonical Wnt signaling function (Hayes et al., 2014). In fact, in contrast to wild-type human PTK7, the hPTK7^P545A^ failed to rescue PCP-dependent axial extension defects as well as nervous system patterning defects caused by Wnt8 overexpression (Hayes et al., 2014). Further, the mutant protein accumulated at the plasma membrane, indicating altered protein stability and/or trafficking of this mutant compared to the wild-type protein. As PTK7 forms co-receptor complexes with Fz7 and LRP6 (Peradziryi et al., 2011; Bin-Nun et al., 2014; Linnemannstons et al., 2014), which were shown to be subject to Wnt-dependent receptor complex trafficking (Yamamoto et al., 2006; Kim et al., 2008; Ohkawara et al., 2011), this is likely also the case for PTK7-containing receptor complexes. Thus, it is tempting to speculate that the proline residue in position 545—which is conserved in mammals—is required for interaction with Wnt ligands or co-receptors, respectively. Interestingly, this conserved P545 residue is also mutated in one of the six sequence variants identified in patients with neural tube closure defects. In a patient affected with myelomeningocele and interestingly also hydrocephalus, which is indicative of a cilia-defect, the non-polar proline residue was changed to a positively charged arginine (Wang et al., 2015). These data indicate that this conserved residue is important for protein function and mutations are likely pathogenic. Future studies are required to elucidate the molecular pathomechanism.

## Conclusions

During the last two decades our understanding of the function of PTK7 has significantly advanced. Diverse biological processes that are regulated by PTK7 have been identified and its role in the establishment of polarity and coordinated cell movements has been acknowledged. Recent publications shed light on a literary “complex” function of PTK7 in Wnt signaling. While its role in non-canonical PCP signaling has been confirmed in different animal model systems and biological contexts, its function with respect to canonical Wnt signaling remains controversial. Possibly, these contradictory findings can be explained by the cell type-specific formation and subcellular localization of distinct co-receptor complexes. Further characterization of the formation and dynamics of these ligand-receptor complexes may help us to understand how PTK7 affects development as well as disease-related processes.

## Author contributions

AB conceptualized and wrote the main part of the paper. AW wrote the *Drosophila* and the cancer section and edited the manuscript; HB wrote the section on scoliosis and designed the two tables.

### Conflict of interest statement

The authors declare that the research was conducted in the absence of any commercial or financial relationships that could be construed as a potential conflict of interest.
